# MoS_2_ nanosheets direct supported on reduced graphene oxide: An advanced electrocatalyst for hydrogen evolution reaction

**DOI:** 10.1371/journal.pone.0177258

**Published:** 2017-05-08

**Authors:** Jiamu Cao, Jing Zhou, Yufeng Zhang, Yuezhang Zou, Xiaowei Liu

**Affiliations:** 1 MEMS Center, School of Astronautics, Harbin Institute of Technology, Harbin, P. R. China; 2 Key Laboratory of Micro-systems and Micro-structures Manufacturing, Ministry of Education, Harbin, P. R. China; Beihang University, CHINA

## Abstract

Molybdenum disulfide nanosheets/reduced graphene oxide (MoS_2_ NSs/rGO) nanohybrid as a highly effective catalyst for hydrogen evolution reaction (HER) have been successfully synthesized by a facile microwave-assisted method. The results clearly reveal that direct grown of MoS_2_ NSs on rGO have been achieved. Electrochemical tests show that the as-prepared hybrid material exhibited excellent HER activity, with a small Tafel slope of 57 mV dec^-1^, an overpotential of 130 mV and remarkable cycling stability. After analysis, the observed outstanding catalytic performance can be attributed to the uniform distribution of the MoS_2_ NSs, which are characterized by the presence of multiple active sites as well as the effective electron transport route provided by the conductive rGO substrate. Moreover, according to the classic theory, the mechanism governing of the catalytic HER on the MoS_2_ NSs/rGO nanohybrid has been clarified.

## Introduction

Hydrogen, a scalable and renewable energy, once produced are environmentally and climatically clean over the entire length of its respective conversion chains, from production to utilization [1]. These advantages are enough to make it be one of the most important energy sources which people rely on after fossil energy [2]. In particular, sustainable hydrogen production from water splitting has attracted growing attention [3]. During hydrogen evolution reaction (HER), advanced catalyst plays an indispensable role, which reducing the overpotential of electrodes and producing a high current density and consequently increasing the yield of this important electrochemical process [4]. Up to now, Pt-group metals still have been enrolled as catalysts in HER. However, the high material costs and limited resource of these catalysts hinder the hydrogen economy [5–7]. Consequently, the ongoing search for efficient alternatives composed of low-cost materials is crucial for a sustainable “hydrogen economy” [8–13].

Molybdenum disulfide is a typical member of transition metal sulfide with a layered structure held together by weak van der Waals forces [14–16]. Recent theoretical calculations and experimental results showed that MoS_2_ to be a competitive electrocatalyst for HER and both computational and experimental date figured the edge sites of MoS_2_ nanoparticles (NPs) are the active sites so that the interest in using MoS_2_ as water-splitting electrocatalysts has intensified [17–20]. However, MoS_2_ exhibits a poor intrinsic conductivity, which severely suppresses charge transport and thus the electrocatalysis efficiency [21,22].

A commonly adopted solution to avoid the above situations is to fabricate nanosized MoS_2_ on a high conductivity substrate [23,24]. Benefiting from its large-sized surface area, the good electrical conductivity, and its stable chemical properties, graphene plane is calculated for acting as a substrate of a composite catalyst [25]. A hybrid catalyst which used reduced graphene oxide (rGO) as the substrate and supported by the MoS_2_ NPs was exhibited the HER catalytic activity with an overpotential of 190 mV and a Tafel slope of 95 mV per dec^-1^ [26]. To further enhance the conductivity of the hybrid catalyst, Cu NPs were incorporated into the MoS_2_/rGO structure, and a decreased Tafel slope of 90 mV dec^-1^ was achieved [27]. But it should be noticed that MoS_2_ NPs are still intended to pile up in quantity as aggregations on rGO. Another potential problem is that a small reduction of rGO will bring a mass of oxygen-containing functional groups on the substrate, leading to decreased of the conductivity. Herein, we report on polymer-free, one-pot, microwave-assisted method for preparing MoS_2_ NSs/rGO nanohybrid by employing ethylene glycol (EG) as reducing agent. We further investigate that the resulting catalyst exhibit unusual catalytic activity in HER.

## Experimental

### Preparation of MoS_2_ NSs

All chemical reagents used in this experiment were analytical grade. The detailed synthesis procedures will be described in the following. Graphene oxide (GO) was prepared following the Hummer's method. A liquid exfoliation technique of ultrasound probe sonication was used to obtain MoS_2_ NSs. This method is less susceptible to the surrounding environment, simple for operation, and suitable for large-scale production. To begin with, 1 g of powder of MoS_2_ was dissolved in 100 mL N-Methylpyrrolidone (NMP), which was placed in a glass vial for a 3.5 h ultrasound under the ultrasound power of 300 W maintaining the temperature at 20°C. Then, the mixture was transferred to centrifuge tubes for the first centrifugal, which was at the speed of 1500 rpm for 60 min and under the temperature at 10°C. The top two-thirds of the supernatant liquid were reserved, and ethanol was added thereto to 300 mL of dilution. After that, the dilution was added into an ultrasonic processor, sonicated with an ultrasound probe for 10 min to obtain the up two-thirds of the solution, and then the solution was centrifuged at 2000 rpm for 60 min. Finally, the supernatant was small-sized MoS_2_ NSs.

### Preparation of MoS_2_ NSs/rGO hybrid material

During the synthesis of the MoS_2_ NSs/rGO hybrid material, 20 mg of GO and (1mg, 3 mg, 5mg) of MoS_2_ NSs were added into 60 mL of a mixture solution of isopropanol and ethylene glycol (v/v = 1:4) and sonicated for 90 min. A 1 M NaOH/EG solution was added to the mixture until a pH of 12 was reached, and then argon was blown into the mixture for 20 min. And then, the mixture was microwaved for ca. 30 s to reach 150°C and was allowed to cool naturally. After that, 1 M dilute nitric acid was added until a pH of 2 was reached. The product was collected by vacuum filtration and vacuum-dried at 60°C.

### Electrochemical evaluation

4 mg of the synthesized MoS_2_ NSs/rGO hybrid material and 80 μL of 5 wt% Nafion were dispersed in 1 mL of a water/ethanol mixture (4:1 v/v) followed by sonication for 15 min to obtain a homogeneous slurry. Subsequently, a glassy carbon electrode (GCE) with a diameter of 3 mm, which was polished by alumina suspensions, was treated with 5 mL of the catalytic slurry and dried naturally. Moreover, pure MoS_2_ NSs and Pt/C modified electrodes were prepared by the same method for comparison purposes. The HER activities of these catalysts were evaluated via linear sweep voltammetry (LSV) in 0.5 M H_2_SO_4_ solution at a scan rate of 5 mV s^-1^ at room temperature (about 26°C). LSV measurements were conducted using an electrochemical workstation (CHI 660D) and a standard three-electrode setup containing a saturated calomel electrode (SCE) as the reference electrode, Pt foil as the counter electrode, and the modified GCEs as working electrodes. Before electrochemical measurements, the polarization curves were corrected for iR losses, the potentials were calibrated using a reversible hydrogen electrode (RHE) at a scan rate of 100 mV/scan, and the utilized electrolytes were degassed by bubbling Ar gas for 1 h. The AC impedance amplitude measured in the frequency range between 105 Hz and 101 Hz with an amplitude of 5 mV. Stable polarization curves were recorded after 2000 cycles.

### Material characterization

The morphology and structure of the MoS_2_ NSs/rGO hybrid catalyst were detected by the transmission electron microscope (TEM) with accelerating voltage at 300 keV. X-ray photoelectron spectroscopy (XPS) was used to record the element composition and the electron binding energy using a K-Alpha (Thermo Fisher Scientific Company) equipment. Energy dispersive spectrometer (EDS) was used to measure the oxygen content with the electron beam being 15 keV. X-ray diffraction (XRD) profiles of the MoS_2_ NSs/rGO hybrid catalyst with high-intensity Cu Kα radiation (λ = 1.5406 nm)in the range of 10°-90°.

## Results and discussions

**Fig 1:**
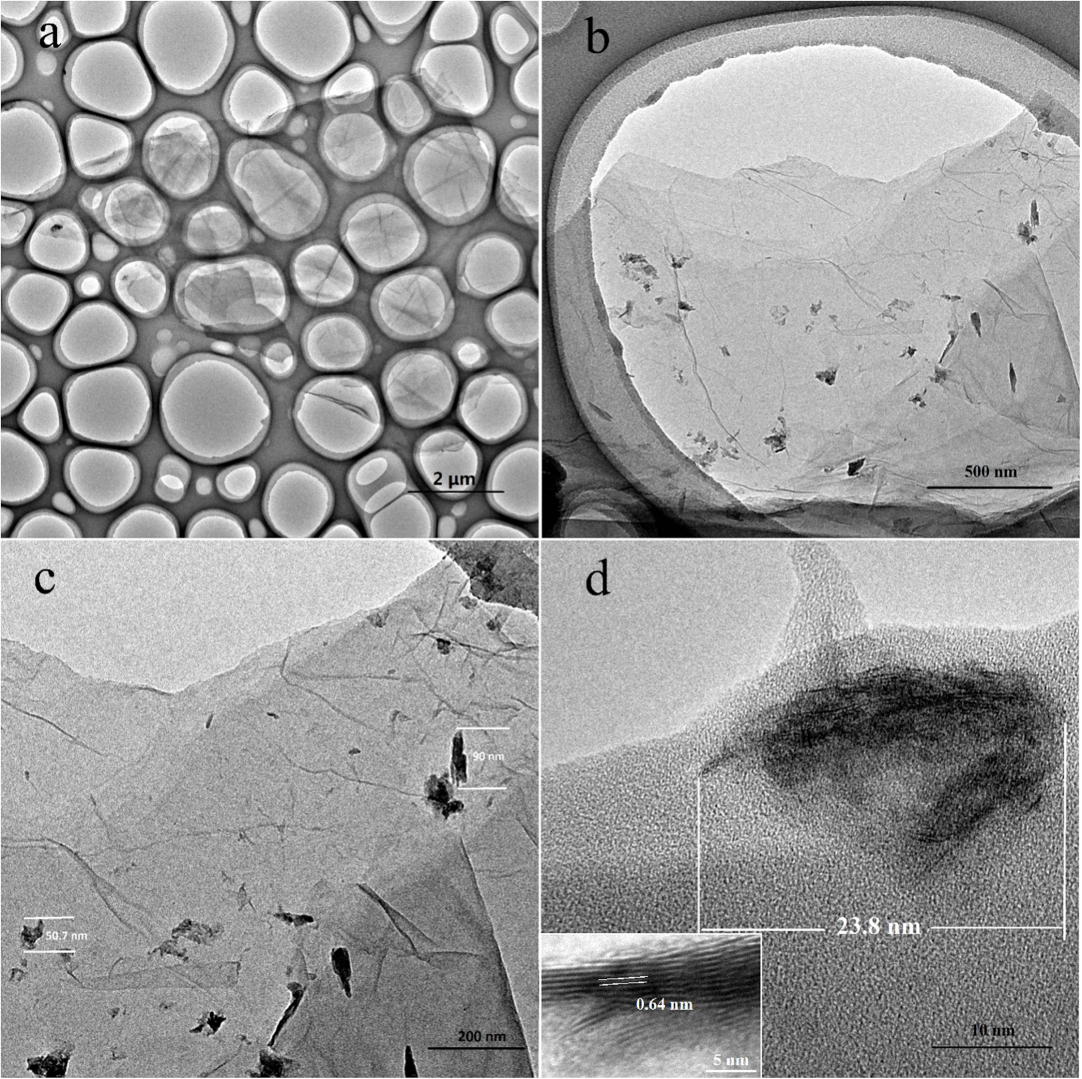
TEM images of MoS_2_ NSs/rGO.

The microstructure of the MoS_2_ NSs/rGO hybrid prepared by microwave assisted method was characterized by TEM, as shown in Fig 1. The micro-sized rGO substrate has a large surface area (Fig 1A). In the magnified image, it can be clearly seen that rGO is very thin and the MoS_2_ NSs with the sizes of 50–90 nm were deposited uniformly on the surface of rGO substrate, remaining isolated from each other (Fig 1B and 1C). The smaller MoS_2_ NS with the length of 23.8 nm and the interlayer spacing of 0.64 nm has plenty edge positions (Fig 1D).

**Fig 2:**
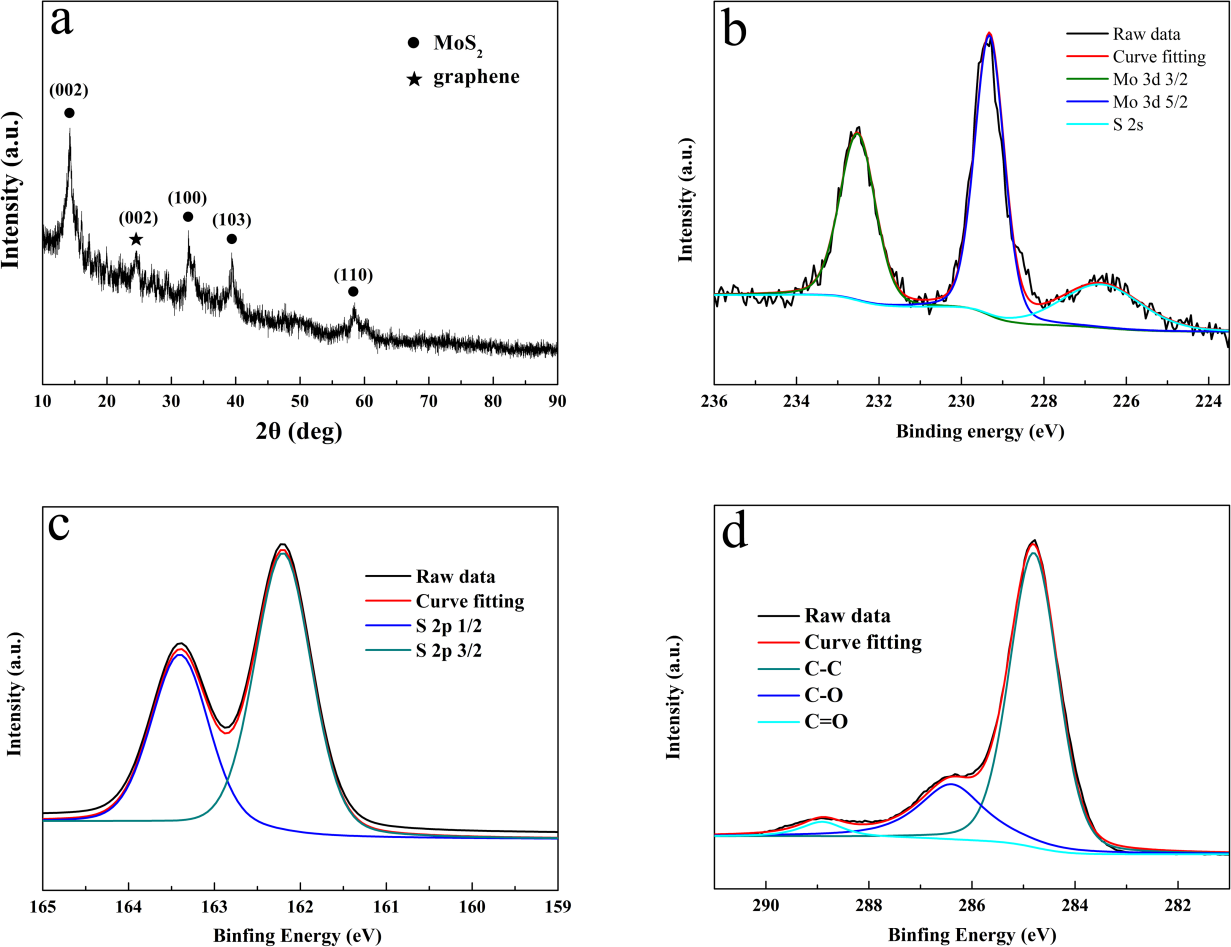
XRD pattern of MoS_2_ NSs/rGO hybrid (a). XPS images of Mo 3d (b) S 2p (c) and C (d).

Fig 2A shows the XRD pattern of the MoS_2_ NSs/rGO hybrid. For the pattern of the sample, the peaks at 2θ = 14.2°, 33.0°, 39.7°, and 59.1° are attributed to the (002), (100), (103) and (110) planes of MoS_2_ [28–29]. Furthermore, we can detect the weak (002) diffraction peaks of the graphene at 2*θ* = 24.5° in the XRD patterns of the hybrids, which indicates that the graphene nanosheets seldom stack during the microwave-assisted process [27]. XPS were used to characterize the chemical states and electronic states of Mo and S in MoS_2_ NSs/rGO hybrid catalyst. As shown in Fig 2B and 2C, the binding energies of Mo 3d_5/2_, Mo 3d_3/2_ peaks at 229.3 eV and 229.3 eV, S 2p_3/2_ and S 2p_1/2_ peaks at 162_._2 eV and 163.4 eV indicate that Mo^4+^ and S^2-^ are the dominant oxidation states. It is obviously to see that there is a slight shift to all the binding energies compared with the reports of pristine MoS_2_ [30]. It is demonstrated that MoS_2_ NSs and rGO substrate was not only simply mixed but also have the interaction which improved its conductivity [31]. Fig 2D shows the XPS spectrum of carbon. The highest peak at 284.8eV represents C−C binding energy. The binding energies of C−O and C = O oxygenated functional groups are located at 286.4eV and 288.9eV.

The relative content of carbon and oxygen indicate the good degree of reduction. The deoxygenating of GO is estimated by C/O radio, which is about 4.9, much higher than that of GO (S1 Table). The C/O ratio was also measured by EDS, as shown in S1 and S2 Figs. The average atomic percent of carbon is about 81.23% and oxygen is about 18.77%. From Chen’s study, the C/O ratio changed from 2.09 to 5.46 after reduction and the reduced GO showed better performance on conductivity, which indicated a high reduction degree [32].

**Fig 3:**
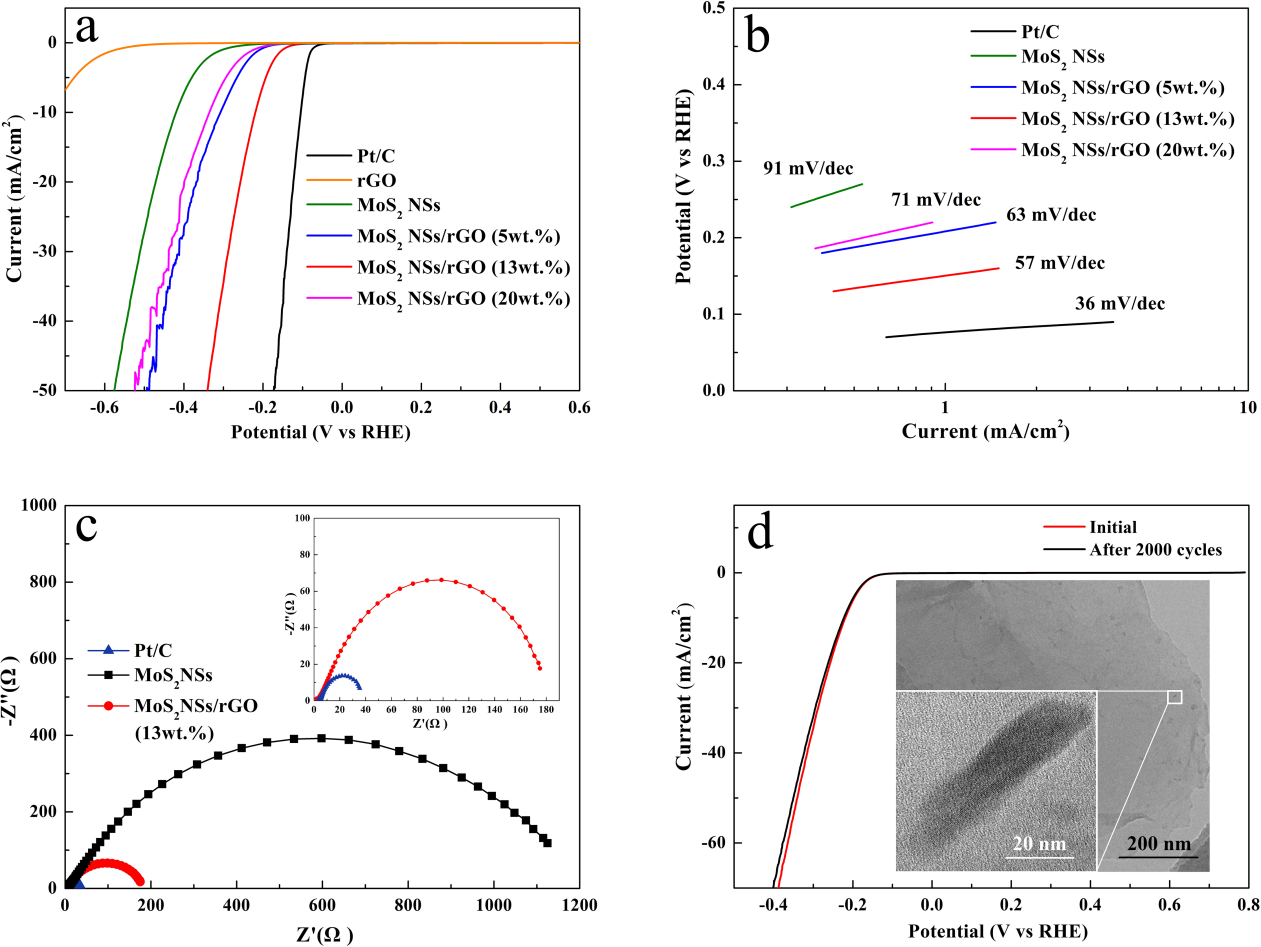
Polarization curves for the catalysts of MoS_2_ NSs, MoS_2_ NSs/rGO (5 wt.%, 13 wt.% and 20 wt.%.), and Pt/C (a) and their corresponding Tafel plots (b). Impedance spectroscopy at an overpotential of 120 mV (c). Durability test for MoS_2_ NSs/rGO hybrid catalyst (d). The inset is TEM image of the MoS_2_ NSs/rGO hybrid after 2000 cycles.

The electrocatalytic HER activities of MoS_2_ NSs/rGO hybrid catalyst (the content of MoS_2_ NSs is identified by atomic absorption spectroscopy as 5 wt.%, 13 wt.% and 20 wt.%) were investigated by polarization curves as shown in Fig 3A, where a commercial Pt catalyst (20 wt.% Pt on Vulcan carbon black) was also included for comparison. It can be seen that the Pt catalyst exhibits very high HER catalytic performance with a near zero overpotential. The as-prepared MoS_2_ NSs/rGO (13 wt.%) hybrid has an overpotential of 130 mV. In sharp contrast, both the rGO and the pure MoS_2_ NSs are exhibited no or poor HER electrocatalytic activities due to their low current densities and large overpotential. The linear segments of the Tafel plots (Fig 3B) were fit to the Tafel equation (*η* = *b*∙lg *j* + *a*, where *j* is the current density and *b* is the Tafel slope), yielding Tafel slopes of 91, 63, 57, 71 and 36 mV dec^-1^ for the MoS_2_ NSs, MoS_2_ NSs/rGO (5 wt.%, 13 wt.% and 20 wt.%.) hybrid and Pt/C. The properties of the as-prepared MoS_2_ NSs/rGO (13 wt.%) hybrid with a Tafel slope of 57 mV dec^-1^ and an overpotential of 130 mV were better than MoS_2_ NSs and those of the MoS_2_/rGO hybrid prepared by previous reports (Tafel slopes of 95 mV dec^-1^ and 90 mV dec^-1^) by the same test method [26, 27].

As a result, the excellent HER performance of the MoS_2_ NSs/rGO (13 wt.%) hybrid catalyst can be attributed to the strong electronic coupling between the MoS_2_ NSs and rGO. To reap this effect, we performed impedance measurements at an overpotential of *η* = 130 mV. As is shown in Fig 3C, in the same amount of catalyst, the MoS_2_ NSs/rGO (13 wt.%) hybrid material exhibits a lower alternating-current impedance of ~180 Ω which very close to that of the Pt/C (alternating-current impedance of ~40 Ω) and much lower than that of the MoS_2_ NSs (alternating-current impedance of~1200 Ω). Another important standard for an excellent electrocatalyst is the high durability. To further evaluate the long-term stability, the MoS_2_ NSs/rGO (13 wt.%) catalyst was cycled continuously for 2000 cycles in an acidic environment. Then, the nanohybrid catalyst afforded similar *i-V* curves just like before, with negligible loss of the cathodic current (Fig 3D). Next, we use TEM to observe that the original morphology of the hybrid was well-maintained (the insert of Fig 3D).

Remarkably, Tafel slopes are one of the most significant factors that can discern the HER mechanism. According to the classic theory [33], Tafel slopes for the typical Volmer, Heyrovsky, and Tafel reactions are around of 120 mV dec^-1^, 40 mV dec^-1^, and 30 mV dec^-1^, respectively (1–3). The following are the now accepted steps by which HER in acidic aqueous media described, where MH_ads_ represents a hydrogen atom chemically adsorbed on an active site of various material (M). In view of the Tafel slope of 57 mV dec^-1^ for the MoS_2_ NSs/rGO (13 wt.%) hybrid in the current work, a combination of the Volmer reaction, involving an electrochemical desorption step that converts protons into absorbed hydrogen atoms on the catalyst surface, and the Heyrovsky reaction, involving the formation of surface scope hydrogen molecules, should dominate the HER on the catalytic process of the MoS_2_ NSs/rGO catalyst. In other words, the rate determining step is the electrochemical desorption of Hads and H_3_O^+^ to form hydrogen, and the HER occurs through a Volmer-Heyrovsky mechanism.

$$H_{3}O^{+}+e^{\text{-}}+C\to MH_{ads}+H_{2}O$$ (1)

$$H_{3}O^{+}+e^{\text{-}}+MH_{ads}\to C+H_{2}+H_{2}O$$ (2)

$$MH_{ads}+MH_{ads}\to 2M+H_{2}$$ (3)

**Fig 4:**
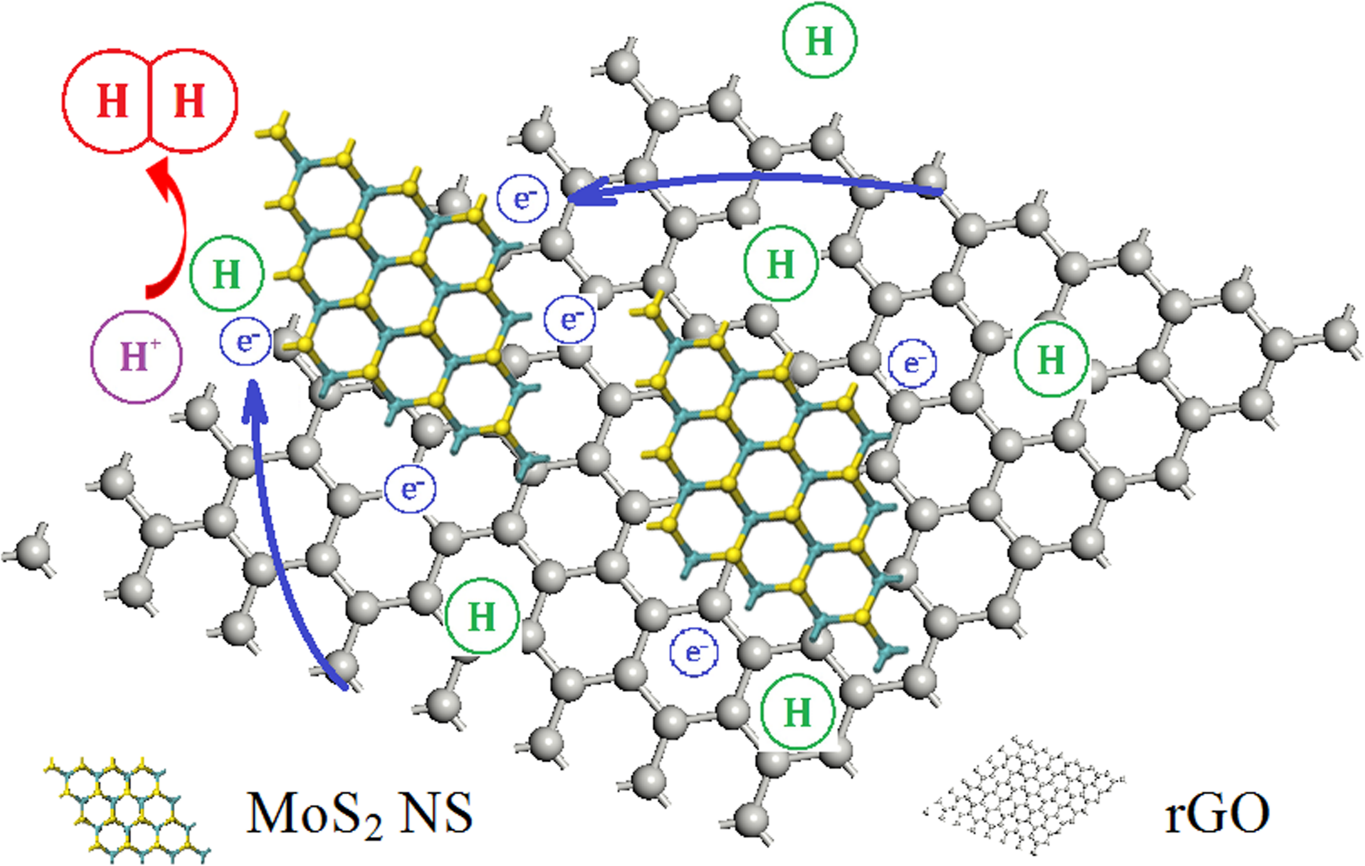
Schematic illustration of the mechanism governing the catalytic HER on the MoS_2_ NSs/rGO structure.

According to the results of our research, the rate-limiting step on the MoS_2_ NSs/rGO catalyst is electrochemical desorption. Fig 4 gives a simple model to demonstrate that synergistic effect of MoS_2_ NSs and rGO in the HER catalytic process are existence. With the rGO functions as substrates, the electrons can be rapidly transferred from C to the active edges of S under an external electric field through the heterojunction. Consequently, the MoS_2_ NSs/rGO hybrid structure can promptly and more efficiently promote the reaction that reduces dissociated H^+^ and produces H_2_ on a fairly large number of active sites.

## Conclusions

In summary, a facile microwave-assisted method has been used to synthesize MoS_2_ NSs/rGO hybrid catalyst for HER. TEM images show that MoS_2_ can disperse better on rGO, which suggests more active sites for HER. Moreover, the MoS_2_ NSs/rGO hybrid catalyst exhibits excellent properties for HER, which may be attributed to the high specific surface area and the uniform distribution of MoS_2_ NSs loaded on rGO. Besides, the distinguished conductivity of rGO and the firm interaction between MoS_2_ NSs and rGO can lead to the parasitic Ohmic losses of the hybrid less than that of original MoS_2_ NSs in HER. Therefore, this work describes an environmentally friendly and low-cost approach to synthesize the effective MoS_2_ NSs/rGO hybrid catalyst, suggesting the great potential applications in HER.

## Supporting information

S1 Fig SEM image of MoS_2_ NSs/rGO (13 wt.%). (DOCX)

S2 Fig The EDS pattern of C and O in MoS_2_ NSs/rGO hybrid catalyst. (DOCX)

S1 Table The test details of XPS (C1s and O1s). (DOC)

## References

[1] Karunadasa HI, Chang CJ, Long JR. A molecular molybdenum-oxo catalyst for generating hydrogen from water. Nature. 2010; 464:1329–1333. doi: 10.1038/nature08969 20428167 10.1038/nature08969

[2] Turner JA. Sustainable Hydrogen Production, Science. 2004; 305:972–974. doi: 10.1126/science.1103197 15310892 10.1126/science.1103197

[3] Luo J, Im JH, Mayer MT, Schreier M, Nazeeruddin MK, Park NG, et al Water photolysis at 12.3% efficiency via perovskite photovoltaics and Earth-abundant catalysts, Science. 2014; 345:1593–1596. doi: 10.1126/science.1258307 25258076 10.1126/science.1258307

[4] Walter MG, Warren EL, McKone JR, Boettcher SW, Mi QX, Santori EA, et al Solar Water Splitting Cells, Chem. Rev. 2010;110:6446–6473. doi: 10.1021/cr1002326 21062097 10.1021/cr1002326

[5] Chen C, Kang YJ, Huo ZY, Zhu ZW, Huang WY, Xin HLL, et al Highly Crystalline Multimetallic Nanoframes with Three-Dimensional Electrocatalytic Surfaces, Science. 2014; 343: 1339–1343. doi: 10.1126/science.1249061 24578531 10.1126/science.1249061

[6] Ye TN, Lv LB, Xu M, Zhang B, Wang KX, Su J, et al Hierarchical carbon nanopapers coupled with ultrathin MoS_2_ nanosheets: Highly efficient large-area electrodes for hydrogen evolution. Nano Energy. 2015; 15:335–342.

[7] Liang X, Zheng HW, Li XJ, Yu YH, Yue GT, Zhang W, et al Nanocomposites of Bi5FeTi3O15 with MoS_2_ as novel Pt-free counter electrode in dye-sensitized solar cells. Ceram. Int. 2016; 42: 12888–12893.

[8] Shi Y, Wang J, Wang C, Zhai TT, Bao WJ, Xu JJ, et al Hot Electron of Au Nanorods Activates the Electrocatalysis of Hydrogen Evolution on MoS_2_ Nanosheets. J. Am. Chem. Soc. 2015; 137: 7365–7370. doi: 10.1021/jacs.5b01732 26020144 10.1021/jacs.5b01732

[9] Ma FK, Wu YZ, Shao YL, Zhong YY, Lv JX, Hao XP. 0D/2D nanocomposite visible light photocatalyst for highly stable and efficient hydrogen generation via recrystallization of CdS on MoS_2_ nanosheets, Nano Energy. 2016; 27:466–474.

[10] Ma LB, Hu Y, Chen RP, Zhu GY, Chen T, Lv HL, et al Self-assembled ultrathin NiCo_2_S_4_ nanoflakes grown on Ni foam as high-performance flexible electrodes for hydrogen evolution reaction in alkaline solution. Nano Energy. 2016; 24:139–147.

[11] Wang M, Chen L, Sun LC. Recent progress in electrochemical hydrogen production with earth-abundant metal complexes as catalysts, Energy Environ. Science. 2012; 5: 6763–6778.

[12] Tang YJ, Wang Y, Wang XL, Li SL, Huang W, Dong LZ, et al Molybdenum Disulfide/Nitrogen-Doped Reduced Graphene Oxide Nanocomposite with Enlarged Interlayer Spacing for Electrocatalytic Hydrogen Evolution. Adv. Energy Mater. 2016; 6:1600116.

[13] Zhou YC, Leng YH, Zhou WJ, Huang JL, Zhao MW, Zhan J, et al Sulfur and nitrogen self-doped carbon nanosheets derived from peanut root nodules as high-efficiency non-metal electrocatalyst for hydrogen evolution reaction. Nano Energy. 2015; 16: 357–366.

[14] Wu WZ, Wang L, Li YL, Zhang F, Lin L, Niu SM, et al Piezoelectricity of single-atomic-layer MoS_2_ for energy conversion and piezotronics. Nature. 2014; 514: 470–474. doi: 10.1038/nature13792 25317560 10.1038/nature13792

[15] Jin BW, Zhou XM, Huang L, Licklederer M, Yang M, Schmuki P. Aligned MoOx/ MoS_2_ Core–Shell Nanotubular Structures with a High Density of Reactive Sites Based on Self-Ordered Anodic Molybdenum Oxide Nanotubes. Angew. Chem. Int. Ed. 2016; 55:12252–12256. 10.1002/anie.201605551 27599478

[16] Yan HH, Song P, Zhang S, Zhang J, Yang ZX, Wang Q. A low temperature gas sensor based on Au-loaded MoS_2_ hierarchical nanostructures for detecting ammonia. Ceram. Int. 2016; 42:9327–9331.

[17] Jaramillo TF, Jorgensen KP, Bonde J, Nielsen JH, Horch S, Chorkendorff I. Identification of Active Edge Sites for Electrochemical H_2_ Evolution from MoS_2_ Nanocatalysts. Science. 2007; 317:100–102. doi: 10.1126/science.1141483 17615351 10.1126/science.1141483

[18] Bonde J, Moses PG, Jaramillo TF, Norskov JK, Chorkendorff I. Paper Hydrogen evolution on nano-particulate transition metal sulphides. Faraday Discuss. 2008; 140:219–231. 19213319 10.1039/b803857k

[19] Hinnemann B, Moses PG, Bonde J, Jorgensen KP, Nielsen JH, Horch S, et al Biomimetic Hydrogen Evolution: MoS_2_ Nanoparticles as Catalyst for Hydrogen Evolution. J. Am. Chem. Soc. 2005; 127:5308–5309. doi: 10.1021/ja0504690 15826154 10.1021/ja0504690

[20] Tiwari AP, Kim D, Kim Y, Prakash O, Lee H. Highly active and stable layered ternary transition metal chalcogenide for hydrogen evolution reaction. Nano Energy. 2016; 28:366–372.

[21] Zhang ZY, Li WY, Yuen MF, Ng TW, Tang YB, Lee CS, et al Hierarchical composite structure of few-layers MoS_2_ nanosheets supported by vertical graphene on carbon cloth for high-performance hydrogen evolution reaction. Nano Energy. 2015; 18:196–204.

[22] Wang QH, Zadeh KK, Kis A, Coleman JN, Strano MS. Electronics and optoelectronics of two-dimensional transition metal dichalcogenides. Nat. Nanotechnol. 2012; 7:699–712. doi: 10.1038/nnano.2012.193 23132225 10.1038/nnano.2012.193

[23] Cao JM, Zhang XL, Zhang YF, Zhou J, Chen YN, Liu XW. Free MoS_2_ Nanoflowers Grown on Graphene by Microwave-Assisted Synthesis as Highly Efficient Non-Noble-Metal Electrocatalysts for the Hydrogen Evolution Reaction. PLOS ONE. 2016; 8: e0161374. 10.1371/journal.pone.0161374 PMC4996486 27556402

[24] Xu S, Lei Z, Wu P. Facile preparation of 3D MoS_2_/MoSe_2_ nanosheet-graphene networks as efficient electrocatalysts for the hydrogen evolution reaction. J. Mater. Chem. A. 2015; 3:16337–16347.

[25] Zhou WJ, Jia J, Lu J, Yang LJ, Hou DM, Li GQ, et al Recent developments of carbon-based electrocatalysts for hydrogen evolution reaction. Nano Energy. 2016; 28:29–43.

[26] Ma C, Qi X, Chen B, Bao SY, Yin ZY, Wu XJ, et al MoS_2_ nanoflower-decorated reduced graphene oxide paper for high-performance hydrogen evolution reaction. Nanoscale. 2014; 6:5624–5629. doi: 10.1039/c3nr04975b 24752376 10.1039/c3nr04975b

[27] Li F, Zhang L, Li J, Lin XQ, Li XZ, Fang YY, et al Synthesis of Cu@ MoS_2_/rGO hybrid as non-noble metal electrocatalysts for the hydrogen evolution reaction. J. Power Sources. 2015; 292:15–22.

[28] Yan S, Qiao W, He X, Guo XB, Xi L, Zhong W, et al Enhancement of magnetism by structural phase transition in MoS_2_. Appl. Phys. Lett. 2015; 106:012408.

[29] Du G, Guo Z, Wang S, Zeng R, Chen Z, Liu H. Superior stability and high capacity of restacked molybdenum disulfide as anode material for lithium ion batteries. Chem. Commun. 2010; 46:1106–1108. 10.1039/b920277c 20126728

[30] Yu X, Du R, Li B, Zhang Y, Liu H, Qu J, et al Biomolecule-assisted self-assembly of CdS/MoS_2_/graphene hollow spheres as high-efficiency photocatalysts for hydrogen evolution without noble metals. Appl. Catal. B-Environ. 2016; 182:504–512.

[31] Suresh C, Mutyala S, Mathiyarasu J, Support interactive synthesis of nanostructured MoS_2_ electrocatalyst for oxygen reduction reaction. Mater. Lett. 2016; 164:417–420.

[32] Chen W, Yan L, Bengal P, Preparation of graphene by the rapid and mild thermal reduction of graphene oxide induced by microwaves. Carbon 2010; 48:1146–1152.

[33] Thomas JGN. Kinetics of electrolytic hydrogen evolution and the adsorption of hydrogen by metals, Trans. Faraday Soc. 1961; 57:1603–1611.

