# Supplementary figures and images for: MoS2 nanosheets direct supported on reduced graphene oxide: An advanced electrocatalyst for hydrogen evolution reaction
Source: PLoS One. 2017 May 8;12(5):e0177258. doi: 10.1371/journal.pone.0177258 (PMC5421784; doi:10.1371/journal.pone.0177258)

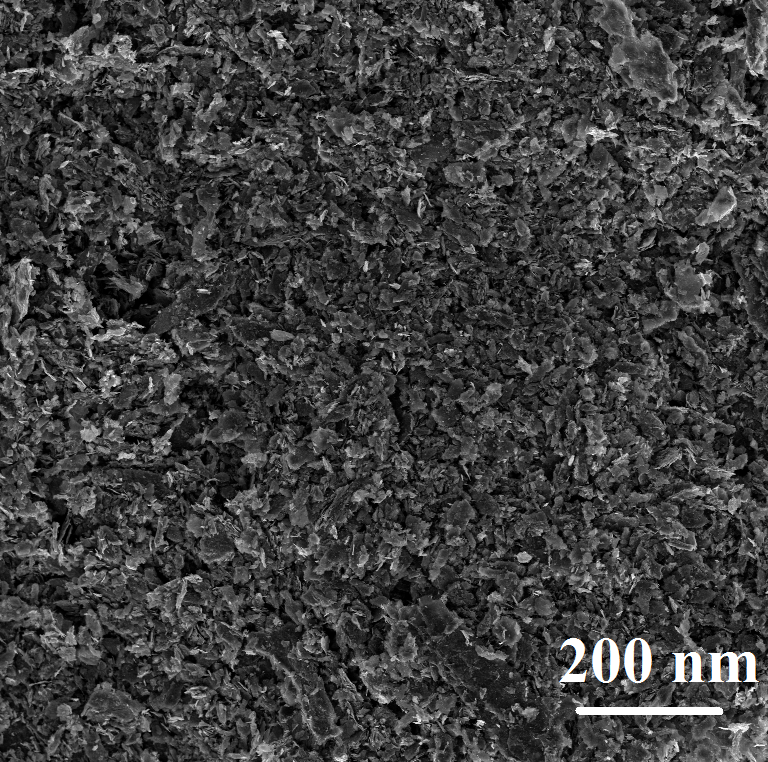


**S1 Fig** SEM image of MoS2 NSs/rGO (13 wt.%)

Supplement: S1 Fig — (DOCX) [file pone.0177258.s001.docx]

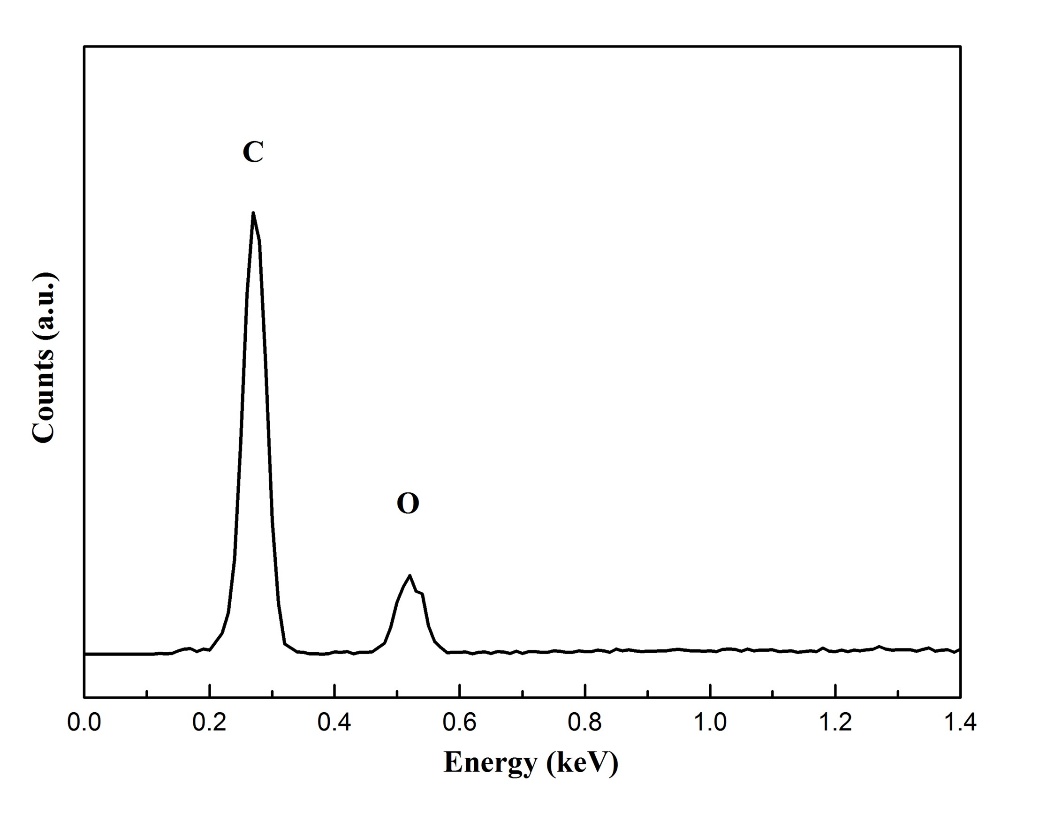


**S2 Fig** The EDS pattern of C and O in MoS2 NSs/rGO hybrid catalyst.

Supplement: S2 Fig — (DOCX) [file pone.0177258.s002.docx]
