# Supplementary material for: MoS2 nanosheets direct supported on reduced graphene oxide: An advanced electrocatalyst for hydrogen evolution reaction
Source: PLoS One. 2017 May 8;12(5):e0177258. doi: 10.1371/journal.pone.0177258 (PMC5421784; doi:10.1371/journal.pone.0177258)
Supplement: S1 Table — (DOC) [file pone.0177258.s003.doc]

**S1 Table** The test details of XPS (C1s and O1s)

| **Name** | **Start Binding Energy (eV)** | **Peak Binding Energy**  **(eV)** | **End Binding Energy**  **(eV)** | **At.%** |
| --- | --- | --- | --- | --- |
| C1s | 298.28 | 284.57 | 279.58 | 83.08 |
| O1s | 545.28 | 532.08 | 525.58 | 16.92 |
